# *Mycobacterium tuberculosis *aortic graft infection with recurrent hemoptysis: a case report

**DOI:** 10.1186/1752-1947-2-233

**Published:** 2008-07-18

**Authors:** Olivier Clerc, Katia Jaton, Guy Prod'hom, Ludwig Von Segesser, Vincent Greloz, Gilbert Greub

**Affiliations:** 1 Infectious Diseases Service, Centre Hospitalier Universitaire Vaudois and University of Lausanne, Switzerland; 2 Institute of Microbiology, Centre Hospitalier Universitaire Vaudois and University of Lausanne, Switzerland; 3 Cardiovascular Surgery Department, Centre Hospitalier Universitaire Vaudois and University of Lausanne, Switzerland; 4 Pathology Department, Centre Hospitalier Universitaire Vaudois and University of Lausanne, Switzerland

## Abstract

**Introduction:**

*Mycobacterium tuberculosis *may cause a large variety of clinical presentations due to its ability to disseminate by contiguity or hematogenously. Tuberculosis may remain undiagnosed for years due to the chronic course of the disease, with potentially life-threatening long-term complications.

**Case presentation:**

In this case report, we describe a tuberculous aortic graft infection in a 72-year-old man documented by polymerase chain reaction and cultures. The patient presented with three episodes of hemoptysis following a remote history of miliary tuberculosis. The infection was treated by graft replacement and prolonged antimycobacterial therapy.

**Conclusion:**

Tuberculous infection of a vascular graft is an uncommon complication, but should be considered in patients with an intravascular device and a history of previous tuberculosis, especially when hematogenous spread may have occurred a few months after surgery, or when an active mycobacterial infection is present in close proximity to the graft.

## Introduction

*Mycobacterium tuberculosis *is a common pathogen that may cause a large variety of clinical presentations due to its ability to disseminate by contiguity or hematogenously. Tuberculosis may remain undiagnosed for years due to the chronic course of the disease, with potentially life-threatening long-term complications. Here, we report a case of mycobacterial aortic graft infection documented by both polymerase chain reaction (PCR) and culture and we review the literature.

## Case presentation

A 72-year-old man with chronic alcohol abuse and hypertension, and who had been a smoker, had had past surgical interventions for atherosclerotic vascular disease (bilateral iliofemoral bypass and abdominal aortic aneurysm repair). In April 2001, the patient presented with severe chest pain due to a descending thoracic aortic aneurysm. At that time, there was no lung abnormality revealed by a computed tomography (CT) scan of the chest. A thoracotomy was performed and a Dacron prosthesis was inserted surgically into the wall of the aneurysm and he had an uncomplicated postoperative course.

Six months later, the patient was hospitalized because of weight loss, night sweats, a dry cough and progressive dyspnea for the past 4 weeks. Chest radiography and CT showed bilateral interstitial lung infiltrates with miliary appearance. No cavitation was documented. A diagnostic bronchoscopy was performed. A Ziehl-Neelsen examination of the lower respiratory tract samples was positive for acid-fast bacilli. PCR and culture of the bronchoalveolar lavage fluid revealed the presence of *M. tuberculosis*. Hematogenous dissemination of *M. tuberculosis *was confirmed by a concomitant positive urine culture. Treatment with rifampicin and isoniazid for 6 months, plus pyrazinamide and ethambutol during the first 2 months was given as directly observed therapy, since the strain isolated in culture was susceptible to all major antimycobacterial drugs.

Three weeks later, the patient presented with massive hemoptysis and severe bronchoaspiration requiring intubation and cardiopulmonary resuscitation. Repeated bronchoscopies documented persistent bleeding from the left lower lobe bronchus. Despite the possible occurrence of a fistula between the aorta and the bronchial tree, the treatment was medical since a chest CT scan did not confirm the presence of a fistula. As bleeding resolved spontaneously, hemoptysis was suspected to be only due to lung tuberculosis and not to aortic involvement. Consequently, the patient was not referred for angiography.

Two months later, the patient presented with a recurrence of hemoptysis of about 300 ml while coughing. Bronchoscopy showed blood clots at the orifice of the left lower lobe bronchus. A CT scan confirmed the hypothesis of an aorto-pulmonary fistula by the presence of a hematoma around the distal anastomosis of the aortic graft and contrast enhancement in the anterior wall of the thoracic descending aorta. Despite the absence of active bleeding at the aortography, an endoprosthesis (Talent type 10 cm) was inserted around the level of the distal anastomosis. Five days later, another bronchoscopy did not show any further bleeding. The bronchial tree appeared normal on endobronchial inspection, and no mycobacteria could be grown from the bronchoalveolar lavage. Consequently, the fistula was not attributed to an uncontrolled infection. Antimycobacterial treatment was continued for about 3 months after this second episode of hemoptysis. The patient was then lost to follow-up and the evolution of the periaortic hematoma could not be monitored.

**Figure 1:**
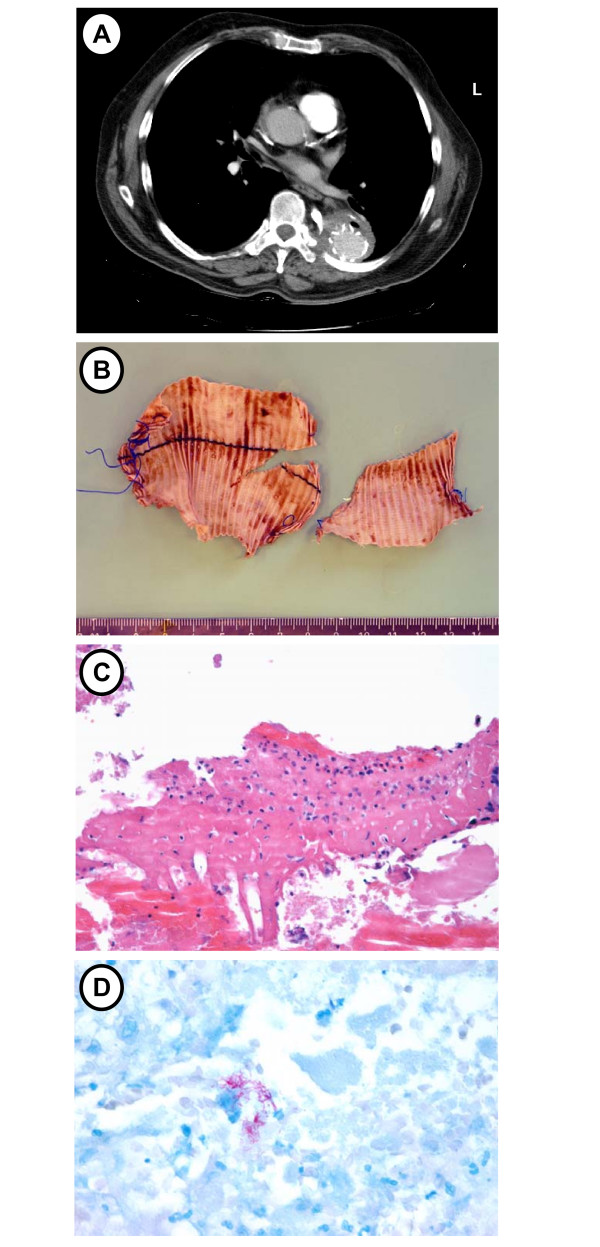
**Radiographic, pathological and microbiological findings**. (A) Computed tomography (CT) showing the presence of an air bubble anterior to the vascular graft, suggesting an aorto-pulmonary fistula. (B) Surgically excised Dacron prosthesis from which *Mycobacterium tuberculosis *DNA was amplified and *M. tuberculosis *grew in culture. (C) Inflammatory cells on a scraping of the prosthesis. No granuloma formation is seen (hematoxylin eosin staining). (D) Ziehl-Neelsen staining of the scraping of the prosthesis showing numerous acid-fast bacilli.

The patient remained asymptomatic until October 2006, when he developed recurrent hemoptysis. CT scan showed air anterior to the vascular graft (Figure 1A), strongly suggestive of an aorto-pulmonary fistula. Bronchoscopy again revealed bleeding originating from the left lower lobe bronchus. A prosthetic infection was suspected. The graft was surgically replaced in November 2006, using a rifampicin-soaked gelatin-sealed polyester graft, after a transaortic debridement of the inside of the aneurysm. At the time of the operation, culture of the bronchoalveolar lavage fluid was still negative for *M. tuberculosis *but turned positive 2 weeks later. On a fragment of the removed prosthesis (Figure 1B), acid-fast bacilli were seen on microscopic examination. Real-time PCR specific for *M. tuberculosis *and culture of the endoprosthesis later returned positive for *M. tuberculosis*. On a scraping of the prosthesis, inflammatory cells were seen (Figure 1C), and Ziehl-Neelsen staining was positive for acid-fast bacilli (Figure 1D). This *M. tuberculosis *infection of the vascular graft, which was responsible for the fistula and secondary hemoptysis, was treated with rifampicin, isoniazid, pyrazinamide and ethambutol. Two months later, given the antibiotic susceptibility of the strain, only rifampicin and isoniazid were continued. The patient's clinical course remained favorable at the last follow-up 9 months after surgery.

## Discussion

Here we report a PCR and culture-proven case of *M. tuberculosis *vascular graft infection that presented with recurrent hemoptysis. The infection may have been due to hematogenous spread or to direct extension from contiguous lung infection. The first hypothesis is supported by the occurrence of a miliary tuberculosis 6 months after aortic aneurysm surgery. However, the second hypothesis is more likely given the localization of lung infection near to the aorta and the documented aortopulmonary fistula.

To the best of our knowledge, only four other cases of *M. tuberculosis *vascular graft infections have been reported to date. The first case was described in 1977 by Wright et al. [1]. Three years after prosthetic aortic graft placement, disruption of the proximal aortic suture and a large pseudo-aneurysm developed. Complete graft excision was required, followed by prolonged antituberculous treatment. Marroni et al. [2] reported the case of an 80-year-old man with fever of unknown origin that was attributed to endovascular aortic graft *M. tuberculosis *infection. Interestingly, the evolution was complicated by an aorto-esophageal fistula. Raffetto et al. [3] described an aortobifemoral Dacron graft infection occurring 2 years after a 6-month course of isoniazid for treatment of latent tuberculosis in an immunocompromised patient who had been treated with high-dose corticosteroids plus infliximab for Takayasu arteritis. A recent report by Sirvanci et al. [4] describes a case of recurrent false aneurysm of the descending thoracic aorta after surgical treatment by excision and patch repair. Infection of the foreign material was not proven.

Aortic aneurysms are rare complications of tuberculosis. From 1950 to 1995, only 41 cases of aortic mycotic aneurysm caused by *M. tuberculosis *have been reported [5]. Approximately 75% of cases were consecutive to a contiguous focus of tuberculosis (lymph nodes, lung, vertebrae or paraspinal abscess) whereas 25% of cases were considered to be of hematogenous origin. Several reports have described hemoptysis consecutive to a bronchial communication with a tuberculous aortic aneurysm [6-8]. The accepted treatment of a tuberculous vascular aneurysm is a combination of surgery and antimycobacterial therapy [9-11]. Neither medical nor surgical therapy leads to cure, if used alone [5]. Recently, it was suggested that endovascular treatment for tuberculous infected aneurysm may lead to life-threatening recurrences, because of the absence of tissue debridement [12]. The optimal duration of treatment is unknown because of the lack of controlled trials. Pre-operative antituberculous therapy may decrease the risk of infection of the implanted graft. Interestingly, medical therapy for tuberculosis might not prevent the development of a tuberculous aneurysm [10].

The case of graft infection reported by Raffetto et al. [3] was successfully managed by performing debulking surgery followed by long-term antimycobacterial therapy. Thus, graft preservation also seems to be possible with long-term suppressive therapy.

Consequently, we decided to treat the patient for at least 1 year after surgery. Whether the treatment might be stopped at that time will be decided based on clinical evolution, inflammatory parameters (C-reactive protein (CRP), leukocyte count) and radiological imaging. After stopping treatment, we propose to measure CRP and leukocyte count regularly and to repeat a chest CT scan twice a year at least for the next 2 years in order to detect a possible recurrence.

## Conclusion

Tuberculous infection of a vascular graft is an uncommon complication, but our case suggests that it should be considered in patients with an intravascular device and a history of previous tuberculosis, especially when hematogenous spread may have occurred a few months after surgery or when an active mycobacterial infection is present in close proximity to the graft.

## Abbreviations

CRP: C-reactive protein; CT: computed tomography; PCR: polymerase chain reaction.

## Competing interests

The authors declare that they have no competing interests.

## Consent

Written informed consent was obtained from the patient for publication of this case report and any accompanying images. A copy of the written consent is available for review by the Editor-in-Chief of this journal.

## Authors' contributions

OC and GG were involved in patient management and wrote the manuscript. OC also reviewed the literature. KJ and GP were involved in the molecular and microbiological diagnosis. LKVS operated on the patient. VG was the supervising pathologist.
